# Supplementary material for: Modulation of DNA Damage Response by SAM and HD Domain Containing Deoxynucleoside Triphosphate Triphosphohydrolase (SAMHD1) Determines Prognosis and Treatment Efficacy in Different Solid Tumor Types
Source: Cancers (Basel). 2022 Jan 27;14(3):641. doi: 10.3390/cancers14030641 (PMC8833711; doi:10.3390/cancers14030641)
Supplement: Supplementary file 1 [file cancers-14-00641-s001.zip › cancers-1568913-supplementary.pdf]

Supplementary tables

Supplementary table S1. Extended clinical data of ovarian cancer patients (*n*=22)

Characteristic	Value
**Age,** years, mean (IQR)	62.82 (51–2)
**Gender,** n (%)	
Female	22 (100%)
**Histology, n (%)**	
High grade serous papillary	17 (77.3%)
Clear cell carcinoma	4 (18.2%)
Low grade serous papillary	1 (4.5%)
**Line of Therapy, n (%)**	
First	1 (4.5%)
Second	7 (31.8%)
Third	6 (27.3%)
≥Fourth	8 (36.4%)
**Type of Therapy, n (%)**	
Cisplatin/ Gemcitabine	1 (4.5%)
Carboplatin/ Gemcitabine	12 (54.5%)
Gemcitabine	9 (41%)
**gBRCA1/2 mutation status, n (%)**	
gBRCA1 and/ or 2 mutated	4 (18.2%)
gBRCA1 and/ or 2 WT	9 (40.9%)
Unknown	9 (40.9%)
**Cause of end of therapy, n (%)**	
Progression	19 (86.4%)
Toxicity (grade 3/4)	3 (13.6%)
**Toxicity grade 3/4, n (%)**	
Yes	10 (45.5%)
No	12 (54.5%)
**Type of toxicity (grade 3/4), n (%)**	
Hematological	8 (80%)
Cutaneous-Mucosal	0 (0%)
Hepatotoxicity	1 (10%)
Other	1 (10%)
**Toxicity consequence, n(%)**	
Dose reduction	1 (10%)
Dose interruption	5 (50%)
Both	4 (40%)

Supplementary table S2. Extended clinical data of breast cancer patients (*n*=46)

Characteristic	Value
**Age,** years, mean (IQR)	52.3 (29–84)
**Gender, n (%)**	
Female	46 (100%)
**Histology, n (%)**	
Invasive Ductal Carcinoma	41 (89%)
Invasive Lobular Carcinoma	3 (6.6%)
Medullar Carcinoma	1 (2.2%)
Metaplastic Carcinoma	1 (2.2%)
**Differentiation grade, n(%)**	
I	1 (2.1%)
II	21 (45.7%)
III	19 (41.3%)
Unknown	5 (10.9%)
**Estrogen Recetor (ER)**	
Negative (<1%)	14 (30,5%)
Positive (≥1%)	32 (69.5%)
**Progesterone Receptor (PR)**	
Negative (<1%)	24 (52.2%)
Positive (≥1%)	22 (47.8%)
**HER2 status#**	
Negative	42 (91.3%)
Positive	4 (8.7%)
**Subtype by immunohistochemistry***	
Luminal A	12 (26.1%)
Luminal B	19 (41.3%)
HER2	4 (8.7%)
Triple negative	11 (23.9%)
**Type of disease**	
De novo or relapsed ≤ 12 momnths	13 (28.2%)
Relapsed > 13 and ≤ 60 months	16 (34.8%)
Relapsed in > 60 months	17 (37%)
**Line of therapy (capecitabine)**	
First	15 (32.6%)
Second	15 (32.6%)
Third	16 (34.8%)
**Cause of end of therapy**	
Progression	44 (95.5%)
Others	2 (4.5%)
**Toxicity grade 3/4, n (%)**	
Yes	15 (32.7%)
No	31 (67.3%)
**Type of toxicity (grade 3/4), n (%)**	
Hematological	1 (6.7%)
Cutaneous-Mucosal	11 (73.3%)
Hepatotoxicity	1(6.7%)

Other	2 (13.3%)
**Toxicity consequence, n(%)**	
Dose reduction	4 (26.7%)
Dose interruption	4 (26.7%)
Both	7 (46.6%)

#HER2 status was evaluated according to the updated 2018 ASCO/CAP guidelines[1]. *Luminal A: ER positive,PR>20%, HER2 negative ki67<14%; Luminal B: ER positive, PR<20%, HER2 negative, and/or ki67≥14%; HER2: HER2 positive; Triple Negative: ER negative, PR negative, HER2 negative.

[1]Wolff, A. C. *et al.* Human epidermal growth factor receptor 2 testing in breast cancer: American society of clinical oncology/ college of American pathologists clinical practice guideline focused update. *J. Clin. Oncol.* **36**, 2105–2122 (2018)

Supplementary table S3. Extended clinical data of lung cancer patients (*n*=16)

**Characteristic**	**Value**
**Age**, years, mean (IQR)	61.58(47–83)
**Gender, n (%)**	
Male	13 (81.3%)
Female	3 (18.7%)
**Line of Therapy**	
First	16 (100%)
**Type of therapy**	
Cisplatin/ Pemetrexed	3(18.8%)
Carboplatin/Pemetrexed	3(18.8%)
Cislatin/ Gemcitabine	5(31.2%)
Carboplatin/ Gemcitabine	4(25%)
Gemcitabine	1(6.2%)
**Type of Presentation**	
De novo metastatic	6 (37.5%)
< 12 months from local therapy	4 (25%)
≥ 12 months from local therapy	6 (37.5%)
**Type of histology, n (%)**	
Scamous	6 (38%)
Adenocarcinoma	5 (31%)
Poorly diferenciated	5 (31%)
**Grade of differentiation**	
I	1 (6.5%)
II	7 (43.5%)
III	8 (50%)
**Cause of end of therapy**	
Progression	2 (12.5%)
End of Terapy	12 (75%)
Exitus	2 (12.5%)
**Toxicity grade 3/4, n (%)**	
Yes	7 (43.7%)
No	9 (56.3%)
**Type of toxicity (grade 3/4), n (%)**	
Hematological	5 (71.4%)
Cutaneous-Mucosal	1(14.3%)
Hepatotoxicity	0(0%)
Other	1 (14.3%)
**Toxicity consequence, n(%)**	
Dose reduction	1 (14.3%)
Dose interruption	5 (71.4%)
Both	1 (14.3%)

Supplementary table S4. Extended clinical data of pancreas cancer patients (*n*=14)

Characteristic	Value
**Age,** years, mean (IQR)	63.57 (45–80)
**Gender, n (%)**	
Male	9 (64.3%)
Female	5 (35.7%)
**Line of therapy (gemcitabine/abraxane)**	
First	13 (92.9%)
Second	1 (7.1%)
**Lines of therapy (post-study)**	
0	4 (28.6%)
1	9 (64.3%)
2	1 (7.1%)
**Performance Status, ECOG**	
0	4 (28.6%)
1	9 (64.3%)
2	1 (7.1%)
**Comorbidity**	
Yes	10 (71.4%)
No	4 (28.6%)
**Type of Presentation**	
De novo	4 (28.6%)
< 12 m	3 (21.4%)
≥ 12 m	7 (50%)
**Cause of end of therapy**	
Progression	10 (71.4%)
Toxicity	4 (28.6%)
**Type of histology, n (%)**	
Adenocarcinoma	14 (100%)
**Toxicity grade 3/4, n (%)**	
Yes	9 (64.3%)
No	5 (35.7%)
**Type of toxicity (grade 3/4), n (%)**	
Hematological	5 (55.5%)
Cutaneous-Mucosal	3 (33.3%)
Hepatotoxicity	0 (0%)
Other	1 (11.2%)
**Toxicity consequence, n(%)**	
Dose reduction	2 (22.2%)
Dose interruption	2 (22.2%)
Both	5 (55.6%)

Supplementary table S5. Extended clinical data of rectum cancer patients (*n*=30)

Characteristic	Value
**Age**, years, mean (range)	62.77(37–80)
**Gender**	
Male	19 (63.3%)
Female	11 (36.7%)
**Performance status, ECOG**	
0	15 (50%)
1	14 (46.7%)
2	1 (3.3%)
**Type of histology, n (%)**	
Adenocarcinoma	30 (100%)
**RDT doses, Gys**	
45	24 (80%)
50	4 (13.3%)
54	2 (6.7%)
**Type of RDT concomitant therapy**	
Capecitabine	30 (100%)
**Type of adjuvant therapy**	
Capecitabine	14 (46.7%)
FOLFOX	7 (23.3%)
XELOX	4 (13.3%)
No Adjuvant therapy	5 (16.7%)

Supplementary table S6. Treatment regimens

Type of tumor	Treatment Squedule
Breast Cancer	**Capecitabine** twice daily at a dose of 2.000 mg/m2/day on days 1-14 in a 3-week cycle
Ovarian cancer	**Gemcitabina** 1,000 mg/m2 on days 1 and 8 and **carboplatin** area under the curve (AUC) 4 mg/mL/min or **cisplatin** 100 mg/m2 on day 1, every 21 days *or* **Gemcitabine** 1000 mg/m2 on day 1,8 every 21 days according of patients *platinum sensitivity or resistance*
NSCLC	**Cisplatin** mg/m2 on day 1 or **Carboplatin AUC 5** on day 1 plus **Gemcitabine** 1,000 mg/m2 on days 1 and 8 *in case of squamous or poorly differenciated carcinoma* *or* **Cisplatin** mg/m2 on day 1 or **Carboplatin AUC 5** on day 1 plus **Pemetrexed** 500 mg/m2 on day 1 every 21 days *in case of adenocarcinoma* *or* **Gemcitabine** 1000 mg/m2 on day 1,8 every 21 days *in case of unfit patients*
Pancreatic Cancer	**nab-paclitaxel** 125 mg/m2 plus **gemcitabine** 1000 mg/m2 (days 1, 8, and 15 of each 28-day cycle)
Early Rectum Cancer	**Induction radiotherapy** *Plus* concurrent to chemotherapy with a daily dose of 1650 mg/m2 **Capecitabine**, divided into two equal doses per day, not including weekends

Supplementary table S7. Effect of different variables and biomarkers in DFI

**Breast Cancer**	**DFI**			
**Variable**	**Univariate**	**P**	**Multivariate**	**P**
**RE**<br><1<br>≥1	.41 (0.20-.082)	**.012**	.66 (.32-1.35)	.25
**Grade**<br>I plus II<br>III	3.47(1.66-7.22)	**.001**	1.9 (.87-4.53)	.10
**SAMHD1**<br><25<br>≥25	3.14(1.55-6.39)	**.001**	2.83 (1.35-5.90)	**.005**
**NSCLC**	**DFI**			
**Variable**	**Univariate**	**p**	**Multivariate**	**p**
**Histology**<br>Scuamous<br>Adenocarcinoma<br>Poorly diferenciated	2.01 (.80-5.03)	.13		
**Diferenciation Grade**<br>I and II<br>III	2.61(.68-9.90)	.15		
**SAMHD1**<br><25<br>≥25	14.71 (1.6-132.66)	**.01**	15.89 (1.09-231.69)	**.04**
**Ovarian Cancer**	**DFI**			
**Variable**	**Univariate**	**p**	**Multivariate**	**p**
**Histology**<br>High grade serous papillary<br>Other	0.27(0.07-0.97)	**.04**	.57 (.11-2.91)	.50
**SAMHD1**<br><25<br>≥25	5.01 (1.40-17.90)	**.01**	3.62 (.78-16.61)	**.098**

Supplementary figures

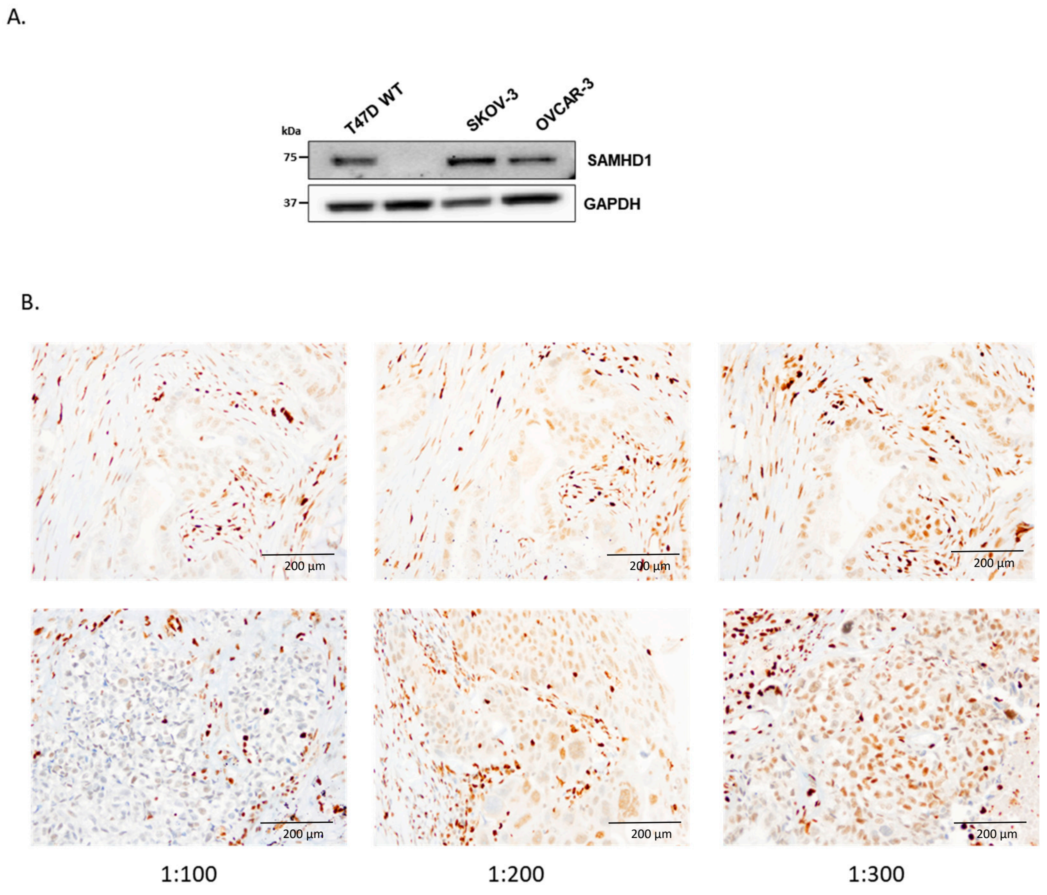

Supplementary figure S1. Validation of SAMHD1 antibody specificity. (A) Detection of SAMHD1 protein in whole cell lysates. Western blot analysis of parental T47D, and two ovarian cancer cell lines (SKOV-3 and OVCAR-3) resulted in a single band at the expected protein size. No SAMHD1 expression was detected in the SAMHD1-KO clone. (B) Formalin-fixed, paraffin-embedded blocks of pancreas (upper panels) and lung (lower panels) cancer samples were prepared and the sections were immunostained with the same SAMHD1 antibody at different antibody concentrations (1:100, 1:200, 1:300). In both cases, a clear strong nuclear staining for SAMHD1 was detected in both tissues, showing a clear dose-response depending on the antibody concentration. All images were obtained at 200x magnification.

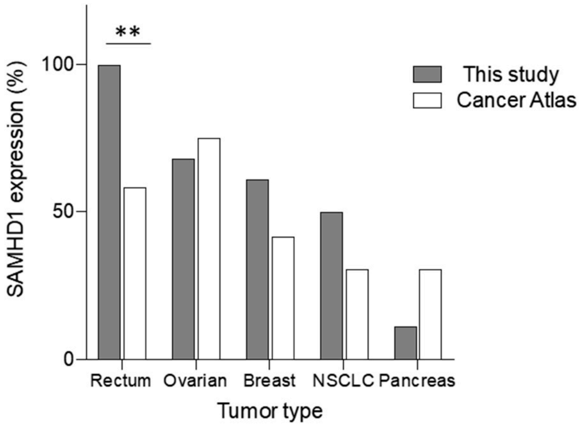

Supplementary figure S2. Comparative analysis of SAMHD1 positivity in distinct cancer cohorts, depending on tumor type. Percentage of positive SAMHD1 tumor biopsies reported in our cohort (grey bars) and in cancer atlas (white bars) in the tumor types tested. No major difference was detected with the exception of rectal tumors (** means significant with a $p < 0.005$).

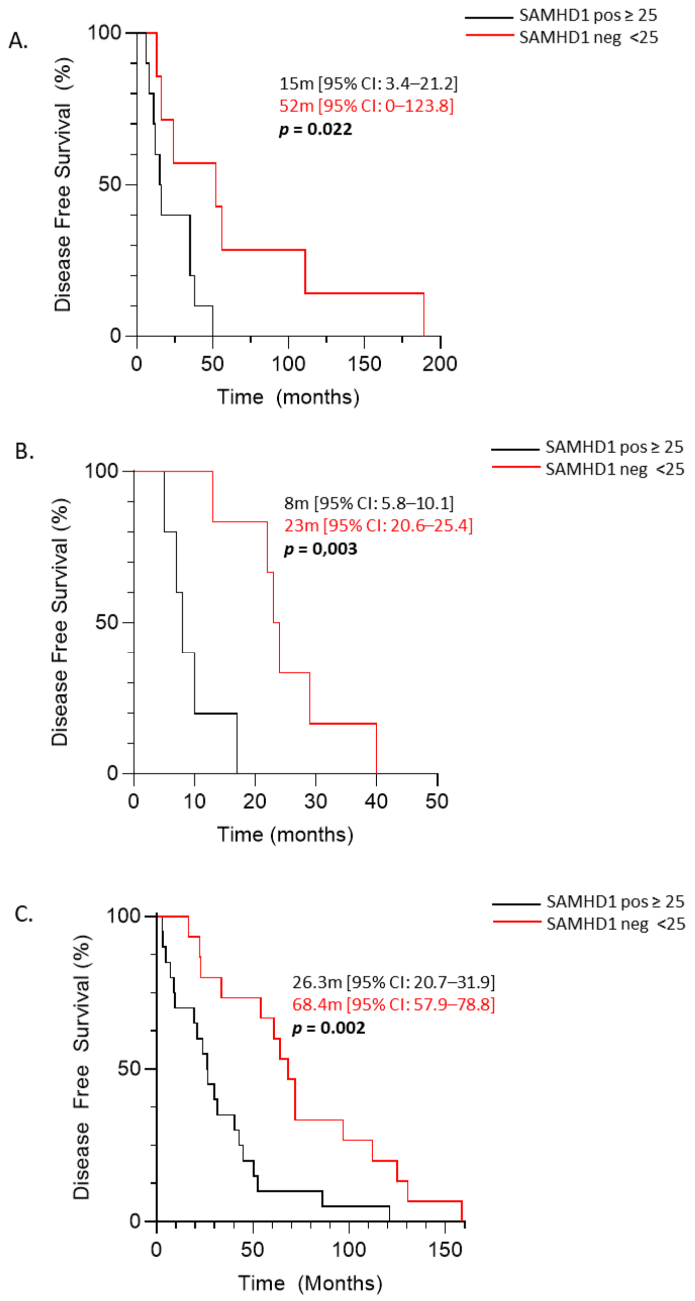

Supplementary figure S3. Kaplan-Meier curves of disease-free survival (DFS) of ovarian (A), NSCLC (B) and breast (C) cancer cohorts stratified by SAMHD1 status. Patients who were diagnosed with advanced disease or received any type of local therapy were excluded from the analysis. SAMHD1 expression below 25% in cancer cells was considered as negative SAMHD1 (red lines) and equal or above 25% was considered as positive SAMHD1 tumors (black lines). Median survival times with CI 95% of both groups are showed. Log rank test was used to test the significance and censored patients are indicated by vertical line.

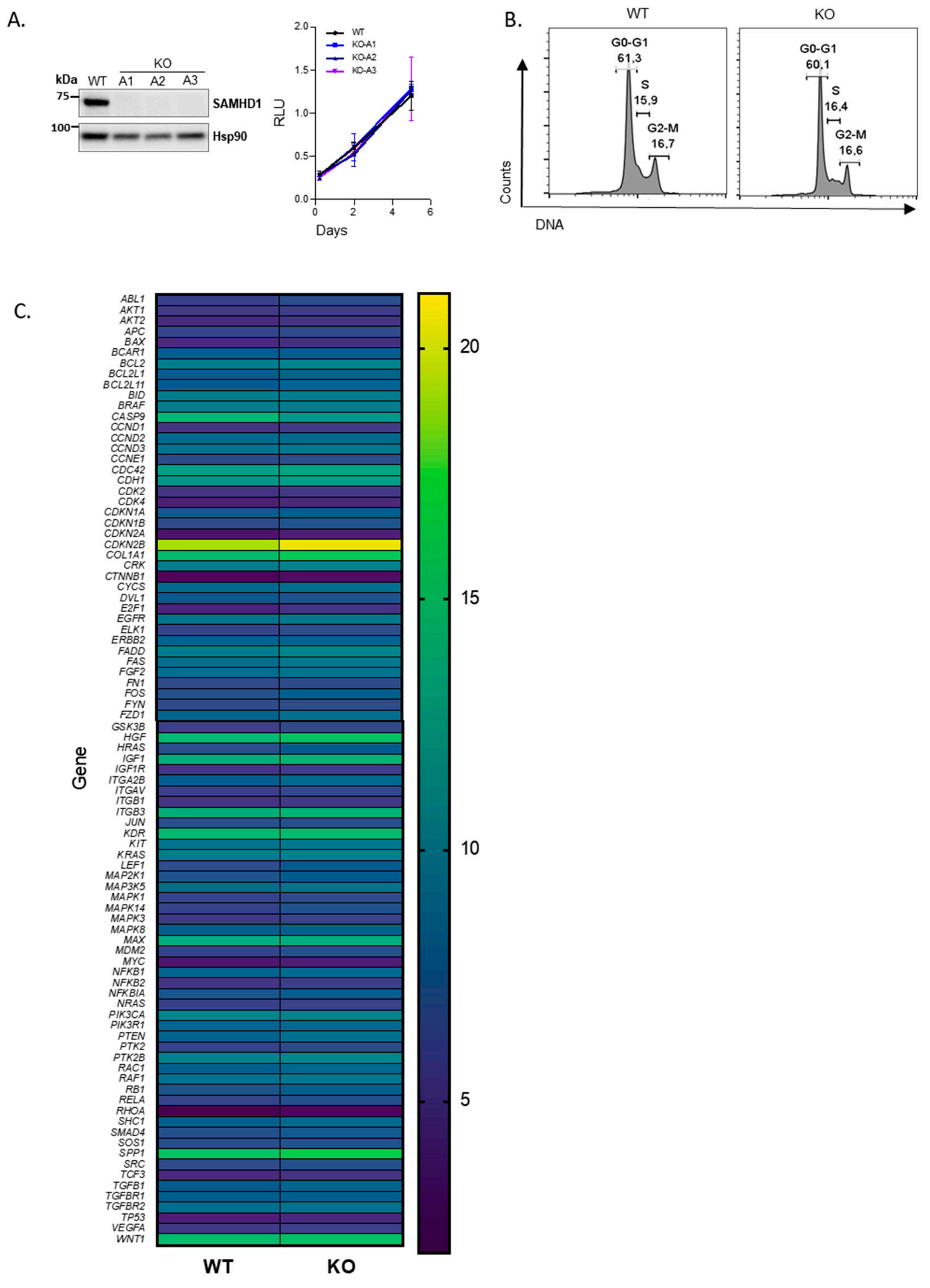

Supplementary figure S4. Characterization of SAMHD1 knockout T47D cells. (A) Representative western blot (left) showing depletion of SAMHD1 in WT and three different clones of SAMHD1-KO T47D cells. Growth curves (right) of wild type (WT, black) and three different clones of SAMHD1 knockout (KO, blue) T47D cells. Cell growth was measured at different timepoints and expressed as relative light units (RLU). Values represent mean ± SD from three different experiments. In WB, Hsp90 was used as a loading control. (B) Cell cycle analysis of WT and SAMHD1 knockout T47D cells. Representative histograms showing relative quantity ratios of G1/G0 phase, S phase and G2-M phase are shown in the figure. (B) Gene

expression profiling in wild-type and SAMHD1 knockout T47D cells. No major differences in main cancer pathways were observed.

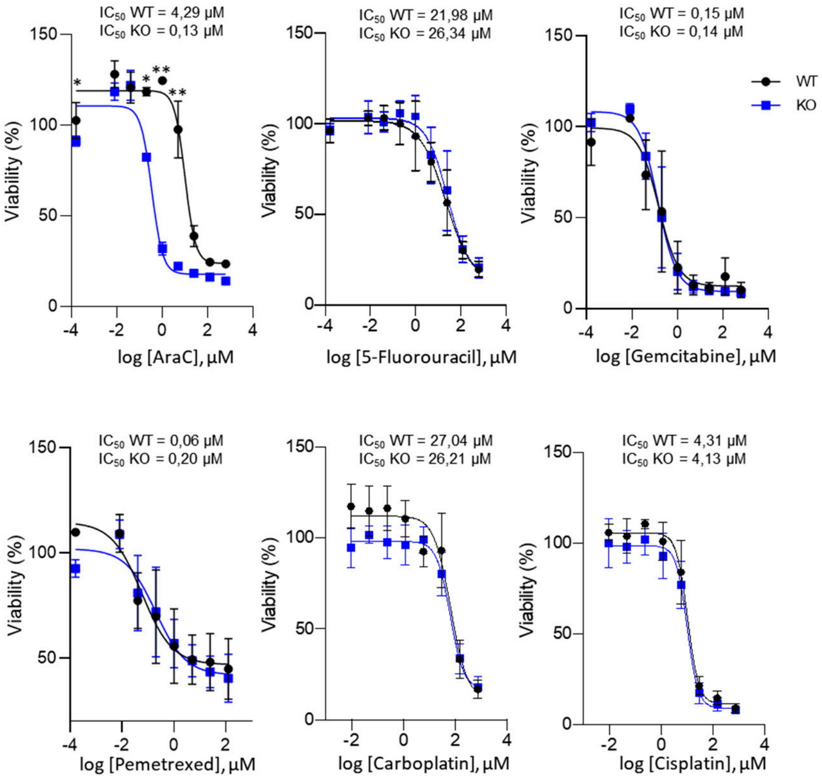

Supplementary figure S5. Dose-response curves showing cell viability of WT (•) and SAMHD1 knockout (■) T47D cells after 4 days of treatment with 5-fluorouracil, gemcitabine, pemetrexed, carboplatin and cisplatin. Cytarabine (AraC) was used as a control. The IC50 values were determined by curve fitting with four parameter non-linear regression analysis. Values represent mean ± SD from three different experiments.

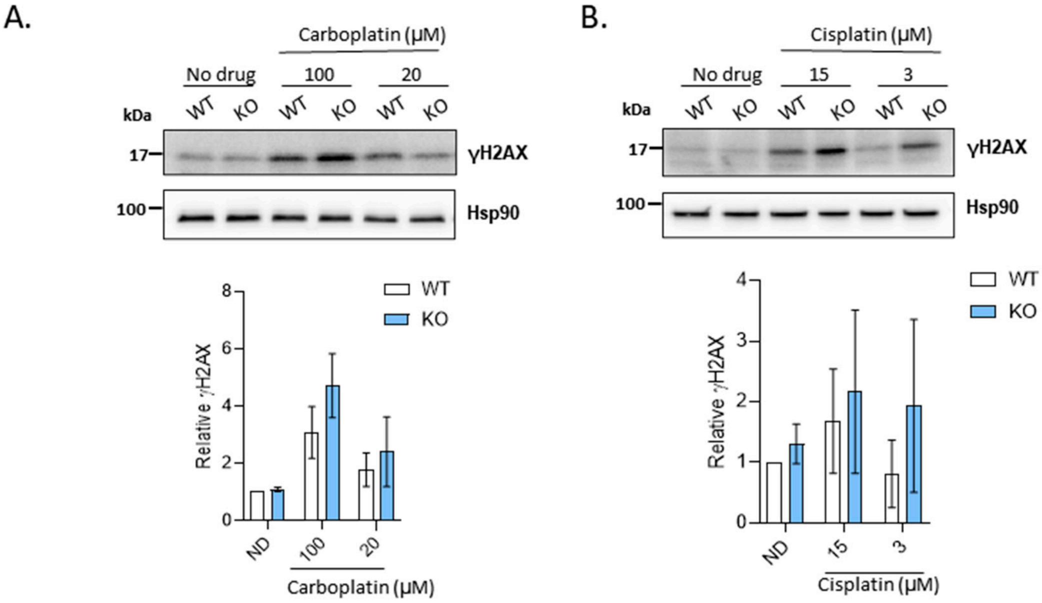

Supplementary figure S6. SAMHD1-depletion induces DNA damage after treatment with platinum derivatives. Representative western blots (upper panel) and quantification (bottom panel) showing γH2AX expression in WT and SAMHD1-KO T47D cells treated with carboplatin (A) and cisplatin (B) for 24h. Mean ± SEM of three different experiments is shown.

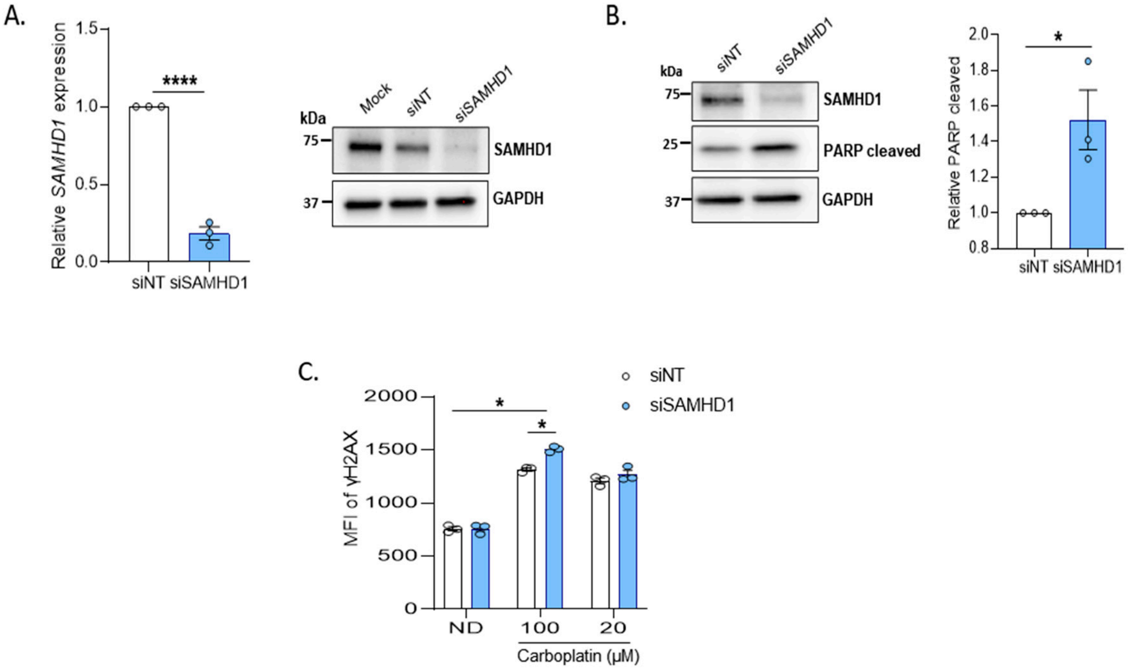

Supplementary figure S7. SAMHD1-depletion induces DNA damage and apoptosis after treatment with platinum derivatives in OVCAR3 cells. (A) SAMHD1 mRNA (left) and protein expression (right) showing specific siRNA-mediated inhibition of SAMHD1 in OVCAR-3 cells. ****, p<0.0001 (B) Representative western blot (left) and quantification (right) showing depletion of SAMHD1 and increased PARP cleaved in SAMHD1-depleted cells. *, p<0.05 (C) Bar graph representing MFI of γH2AX expression in siNT and siSAMHD1 OVCAR-3 cells after treatment with different concentrations of carboplatin. Mean ± SEM of three different experiments is shown. *, p<0.05.

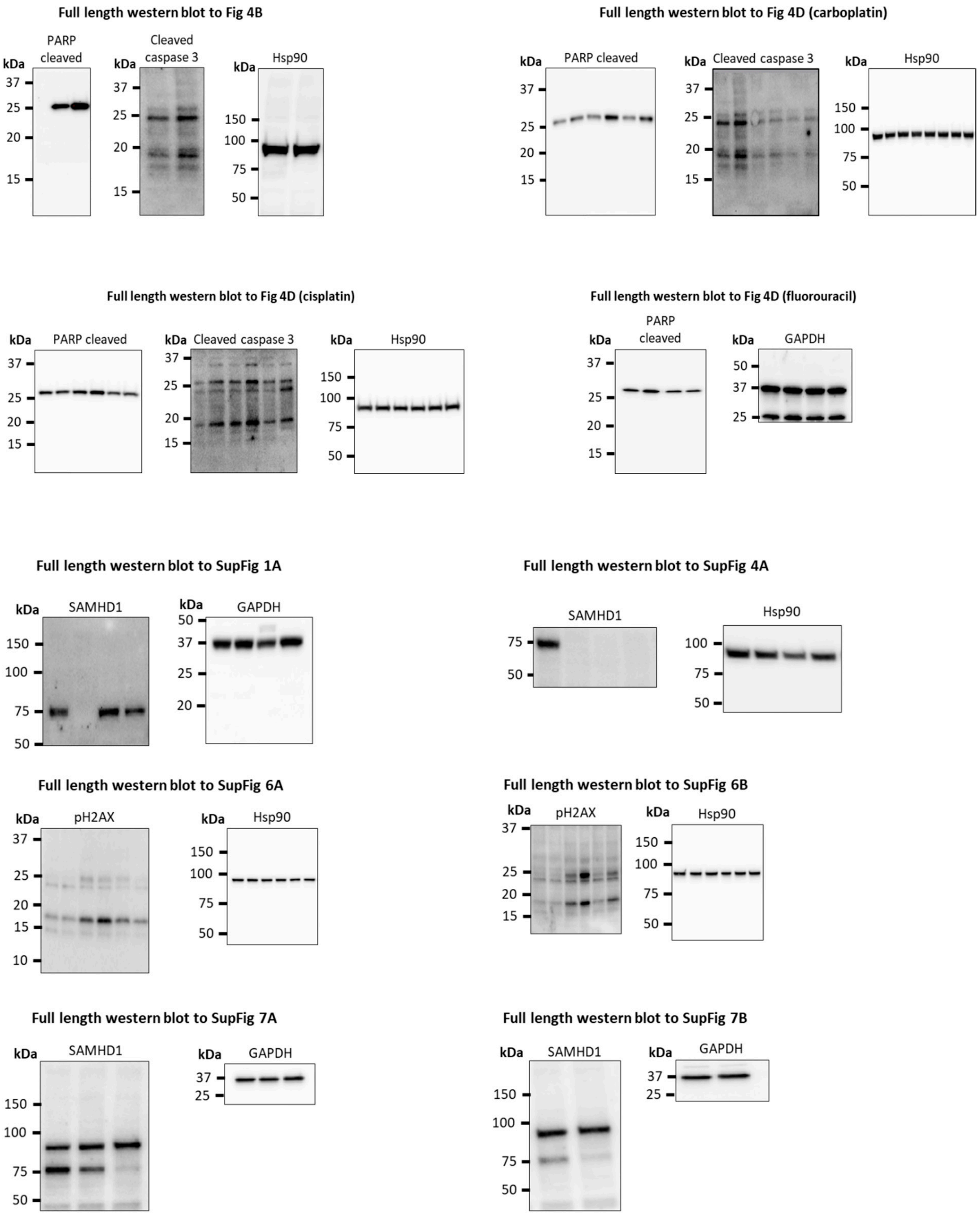

Supplementary figure S8. Full length western blots.
